# Diagnostic and prognostic values of B-type natriuretic peptides (BNP) and N-terminal fragment brain natriuretic peptides (NT-pro-BNP)

**DOI:** 10.5830/CVJA-2013-055

**Published:** 2013-10

**Authors:** Lorena Maries, Ioan Manitiu

**Affiliations:** Lucian Blaga University, Sibiu, Romania; Lucian Blaga University, Sibiu, Romania

**Keywords:** natriuretic peptides, prognostic values, NT-pro-BNP

## Abstract

**Abstract:**

B-type natriuretic peptide (BNP) is a member of a four-natriuretic peptide family that shares a common 17-peptide ring structure. The N-terminal fragment (NT-pro-BNP) is biologically inert, but both are secreted in the plasma in equimolar quantities and both have been evaluated for use in the management of congestive heart failure. BNP and NT-pro-BNP are frequently used in the diagnosis of congestive heart failure and distinguishing between patients with dyspnoea of cardiac or pulmonary origin. Values of NT-pro-BNP are affected by age or the presence of one or several co-morbidities such as chronic renal failure, type 2 diabetes, and acute coronary syndrome. ‘Normal’ values of these peptides also vary depending on the type of test used. The performance characteristics of these tests vary depending on the patients on whom they are used and the manufacturer. For this reason, the determination of reference values for this peptide represents such a challenge.

## Abstract

BNP was initially discovered in the porcine brain, but the largest concentrations are found in the heart. It is a peptide with 32 amino acids, synthesised in the ventricles as a response to stretching of the myocytes and/or pressure overload. It is released as an active hormone and as an inactive N-terminal fragment (NT-pro-BNP).^1^

Once released in the blood flow, BNP has numerous physiological actions, their net effect being to reduce pre- and post-load. Specifically, BNP produces a decreased vascular tonus by relaxing the smooth muscles, leading to a decrease in post-load. In addition, it induces a movement of fluid into the interstitial space, thus leading to a decrease in pre-load.

BNP reduces the proliferation of fibroblasts and smooth muscle cells, sympathetic nervous activity, water and salt retention, release of the antidiuresis hormone, and synthesis of aldosterone and its release from the adrenal glands. In the kidneys, BNP increases glomerular filtration rate and renal blood flow by increasing the outgoing arterial tonus and decreasing the ingoing one. In addition it decreases the release of renin and the reabsorption of sodium, leading to diuresis and natriuresis.^2^

The N-terminal fragment of BNP is derived from proteolysis of pro-BNP, which is composed of 108 amino acids. It consists of 76 amino acids and has recently caused great interest, due to its possible role in monitoring heart failure and distinguishing acute coronary syndromes. Its effects on diuresis and natriuresis in patients with congestive heart failure represent a compensatory mechanism for stress on the myocytes, which leads to ventricular dysfunction.^1^

## Diagnostic and prognostic value of BNP and NT-pro-BNP

Serum levels of natriuretic peptides are important, not just as indicators of numerous cardiovascular deficiencies but also as markers of their severity.1 For patients with acute coronary syndromes, the determination of BNP levels offers predictive information on the apportioning of risk, in the absence of elevation in the S-T interval. In addition, BNP and NT-pro-BNP have prognostic significance for acute pulmonary embolism.^1^

The diagnostic value was recently confirmed by Coutance *et al.*^3^ Even if high levels of BNP demonstrate a high sensitivity for detecting patients with risk of sudden death, the specificity of this neurohormone is decreased. A diverse analysis between mortality and levels of BNP was recently conducted by Nunez and his team, which demonstrated a positive linear correlation between the risk of death and BNP level.^4^

With regard to the prognostic value of NT-pro-BNP for chronic heart failure, the Val-HeFT study (Valsartan Heart Failure Trial) demonstrated the positive nature of advanced heart failure. Moreover, BNP concentrations appeared significantly increased in patients with dilated cardiomyopathy and cardiovascular disease in NYHA classes III or IV, but it could not predict mortality or the requirement for a heart transplant.^4^

## Variability of BNP

Despite the evidence that BNP is secreted in ventricular overload states, there is an individual and inter-individual variation in both healthy subjects and those with stable chronic heart failure, which makes the interpretation of BNP levels difficult. Multiple studies have shown that only changes in BNP level larger than approximately 113 to 130% and changes in NT-pro-BNP larger than 90 to 98% can be considered to have exceeded individual, inter-individual and analytical variations.^2^

There are several reasons for these variations. In healthy subjects, BNP level is connected to gender and age; its levels increase with age and are higher in women than in men. Despite increases with age, BNP and NT-pro-BNP proved effective in excluding congestive heart failure in an elderly population that presented with acute dyspnoea. Race also plays a role, with a higher variability being seen in African-Americans than Caucasians.^2^ Studies conducted in Africa found reference values higher than those recommended by the manufacturer.^5^ A recent study showed that the reference value for NT-pro-BNP depends on age over 50 years.^6^ Another study containing nonagenarian patients reported a link between values of NT-pro-BNP and echocardiographic anomalies.^7^

Levels of BNP are lower for obese patients compared to non-obese. In addition, it was observed that genetics plays a role in the variability of BNP levels. Along with age, gender, genetics and body mass index, there are other physiological reasons for the variability of BNP.

Renal function also affects levels of BNP, significantly increased levels being recorded for those with renal dysfunction. Patients on haemodialysis showed significant rhythmic oscillations in BNP levels, compared to healthy subjects.^2^ In the Breathing Not Properly study, BNP predictors from ‘the grey area’ in the absence of heart failure included age, atrial fibrillation, lower body mass index and anaemia.^2^

The lack of a single set of normal values due to different idiopathic levels and the available commercial kits can lead to confusion in clinical application. While some researchers claim that values above 100 pg/ml indicate heart failure, others suggest a value above 200 pg/ml. The ADHERE study, which included over 48 000 patients, indicated as prognosticators of mortality values over 430 pg/ml.^8^ In all cited conditions, a careful clinical examination, accompanied by an echocardiographic examination that evaluates the systolic–diastolic function, should be complementary to BNP analysis for diagnostic strategy and implementation of treatment.^9^

## Heart failure

Chronic heart failure is an illness that is appearing with increasing frequency, especially in elderly patients. Nevertheless, classification is often difficult due to non-specific symptoms and the lack of a ‘gold standard’ protocol for a correct diagnosis.^9^ The European guidelines from 2008 highlight the role of natriuretic peptides as potential markers of heart failure.^9^

Measurement of plasma concentrations of BNP has proved to be a very efficient screening technique for the identification of patients with various heart diseases, regardless of aetiology and the degree of systolic dysfunction of the left ventricle, which has the potential to develop into manifested heart failure and has a high risk of producing a cardiovascular event. Recently, the Food and Drug Administration approved NT-pro-BNP for evaluation of the prognosis of patients with congestive heart failure and acute coronary syndromes. Determination of BNP level was also approved for risk segregation in acute coronary syndromes.^10^

Multiple studies have confirmed the efficiency of the determination of BNP concentrations in the plasma of patients with acute dyspnoea. The Breathing Not Properly study is an example, in which 1 586 patients participated.^9^ In addition, studies such as Val-HeFT^11^,^12^ and COPERNICUS^13^ indicated that chronic treatment with beta-blockers and blockers of the renin–angiotensin–aldosterone system led to a reduction in levels of natriuretic peptides in the plasma and improved the prognosis, which was possibly a reflection of the improvement in cardiac function secondary to treatment.^2^

Together with its role in acute decompensated heart failure, levels of BNP are also high for diastolic dysfunction. Increased BNP levels can be found with isolated diastolic dysfunction, hypertrophic cardiomyopathy, or associated with systolic dysfunction. Echocardiographic parameters correlated with BNP levels include mass index of the left ventricle, its end-diastolic volume and isometric relaxation time. The further the stage of diastolic dysfunction the higher the levels of BNP.^2^

## Other heart diseases

As with congestive heart failure, BNP level has a prognostic value for acute coronary syndromes. BNP is additive with, and independent of, the increases in troponin I for these syndromes.^2^

A sub-study of Breathing Not Properly showed that plasma levels of BNP were high for patients with atrial fibrillation that was not diagnosed with congestive heart failure, but its levels were not different in the presence of heart failure.^2^ In addition, levels of BNP were high with heart valve diseases and aortic stenosis, and were linearly related to the symptoms. Moreover, levels over 190 pg/ml foresaw a negative evolution, suggesting that BNP can be used for identification of subgroups of patients that would benefit from a replacement of the aortic valve. In addition, BNP level was increased with aortic insufficiency.^2^

For patients with mitral insufficiency, an increased BNP level was correlated with mortality and the onset of congestive heart failure, regardless of the degree of regurgitation present on echocardiography, suggesting that BNP is a reflection of its atrial and ventricular consequences.^2^ Finally, it was proven that NT-pro-BNP was correlated with symptoms and echocardiographic severity of mitral stenosis.^2^ In addition, the levels of BNP were increased in patients with pulmonary embolism and pulmonary hypertension.^2^

In unstable angina, NT-pro-BNP represents an effective marker of the damage produced by cardiac ischaemia. The severity of the coronary disease is shown by an increase in the levels of NT-pro-BNP. In addition, in the case of acute coronary syndromes, NT-pro-BNP had an immuno-modulating role and offered important information for the prognosis of patients.^1^

Castro *et al*.^14^ divided 87 patients with non-ST-segment elevation acute coronary syndrome into two groups: 37 (42.5%) with unstable angina and 50 (57.5%) with non-ST-segment elevation myocardial infarction. Left ventricular ejection fraction above 40% was found in 86.2% of the total sample. Serum levels of NT-proBNP were higher in patients with non-ST-segment elevation myocardial infarction than in those with unstable angina (*p* < 0.001).^14^

Increased levels of NT-pro-BNP were associated with increases in troponin I (rs = 0.425, *p* < 0.001), peak CK-MB (rs = 0.458, *p* < 0.001) and low left ventricular ejection fraction (rs = –0.345, *p* = 0.002); no correlation was found with the TIMI risk score (rs = 0.082, *p* = 0.44). Multivariate analysis revealed that left ventricular ejection fraction and troponin I levels were independently correlated with NT-pro-BNP levels (*p* = 0.017 and *p* = 0.002, respectively).^14^

## Renal failure

Renal failure complicates congestive heart failure so often that many have suggested a ‘cardio–renal’ syndrome, which influences survival, duration of hospitalisation and re-admission ratio.^2^ A sub-study of PRIDE^15^ showed a reduction in the sensitivity and specificity of NT-pro-BNP in the diagnosis of heart failure for persons with renal failure, and also showed that its concentration tends to be more affected by renal dysfunction than BNP levels.^2^ The levels of BNP are known to be significantly increased for patients on haemodialysis, and they are known to decrease after dialysis.^2^

In another study that involved 72 patients on haemodialysis, NT-pro-BNP level was not associated with heart failure, but was dependent on factors associated with an increase in post-load.16 An association between increased levels of NT-pro-BNP and chronic renal failure was also demonstrated in patients without left ventricular dysfunction.^17^,^18^

## Diabetes mellitus

In a study on 371 patients with heart failure, 81 of whom had diabetes, the levels of 10 neurohormones from the plasma (adrenaline, noradrenaline, dopamine, aldosterone, renin, endothelin, ANP, NT-pro-ANP, BNP and NT-pro-BNP) were measured. All patients were also part of the PRIME-II study that investigated the effects of ibopamine on the causes of mortality in patients with moderate or severe heart failure.^19^

Most of the neurohormones were similar between the two groups, but patients with diabetes had higher values of BNP and NT-pro-BNP. The patients were monitored for five years, and during this time, 195 died, of whom 51 had diabetes. For patients with diabetes, noradrenaline, ANP, NT-pro-ANP, BNP and NT-pro-BNP levels were significantly higher than in those who did not survive. Therefore BNP and NT-pro-BNP proved the strongest predictors of outcome for both groups of patients.^19^

The most likely explanation for the increase in BNP and NT-pro-BNP levels in these patients with diabetes was the presence of diastolic dysfunction.^19^ Another study showed normal values of NT-pro-BNP for women with gestational type 2 diabetes mellitus, and lower values for those with insulin-dependent gestational diabetes.^20^

## Cirrhotic cardiomyopathy

Cirrhotic cardiomyopathy is an under-diagnosed condition. This is most likely due to the fact that there is no single diagnostic test to identify these patients.^21^

Numerous recent studies demonstrated that patients with hepatic cirrhosis had increased plasma concentrations of BNP and NT-pro-BNP, representing markers of early ventricular dysfunction. Henriksen *et al*.^22^ showed that these markers were correlated with the severity of hepatic cirrhosis, and with heart dysfunction. BNP could therefore have prognostic value with regard to the evolution of cirrhosis. In addition NT-pro-BNP represents a useful marker to demonstrate the existence of diastolic dysfunction of the left ventricle caused by a chronic hepatic disease.^23^

A study conducted on 153 patients subjected to a liver transplant determined their BNP levels post-transplant and on days 1 and 7. It was observed that a BNP level higher than 391 pg/ml immediately after the liver transplant appeared to be an early marker for heart dysfunction related to the cirrhosis.^24^

## Conclusion

In patients with dyspnoea, overlapping or even conflicting history, physical and radiographic findings often hinder the differentiation between cardiac and non-cardiac aetiology. The primary value of BNP and NT-pro-BNP testing in the emergency department is its diagnostic value in the differential diagnosis of acute dyspnoea and possible congestive heart failure.

Levels of natriuretic peptides may also assist the emergency physician in appropriately triaging the patient with congestive heart failure.^25^ Studies have shown that measurements of BNP or NT-pro-BNP in the emergency department can be used to establish the diagnosis of congestive heart failure when clinical presentation is ambiguous or when confounding co-morbidities are present.^25^

After multiple studies, the conclusion was reached that levels of BNP < 100 pg/ml and > 500 pg/ml have a positive and negative predictive value, respectively, of 90% for the diagnosis of congestive heart failure for patients presenting with acute dyspnoea. For values between 100 and 500 pg/ml, the physicians must consider underlying left ventricular dysfunction, the effects of renal failure, or right ventricular dysfunction secondary to chronic pulmonary disease or acute pulmonary embolism.^25^

The recommended thresholds of less than 100 pg/ml to rule out heart failure and more than 500 pg/ml to rule in heart failure have been estimated to have the following likelihood ratios (LRs): LR-negative = 0.13 and LR-positive = 8.1. These different cut-off values create an intermediate range of 100–500 pg/ml with an LR-positive of only 1.9 pg/ml. Therefore, an intermediate BNP result alone cannot be used to rule in or rule out heart failure.^25^

## References

[1] Cacciaputo F (2010). Natriuretic Peptide System and Cardiovascular Disease.. Heart Views.

[2] Burke MA, Cotts WG (2007). Interpretation of B-type natriuretic peptide in cardiac disease and other comorbid conditions.. Heart Fail Rev.

[3] Coutance G, La Page O, Lo T, Hamon M (2008). Prognostic value of brain natriuretic peptide in acute pulmonary embolism.. Critical Care.

[4] Nunez J, Nunez E, Robies R, Bodi V, Sanchis J, Carratala A, Aprici M, Liacer A (2008). Prognostic value of brain natiuretic peptide in acute heart failure: mortalità and hospital redmission.. Rev Esp Cardiol.

[5] Ajuluchukwu J, Ekure E, Mbakwem A (2010). Reliability and accuracy of point-of-care amino-terminal probrain natriuretic peptide in congestive heart failure patients.. Internet J Cardiol.

[6] Bernstein LH, Zions MY, Alam ME (2011). What is the best approximation of reference normal for NT-pro-BNP? Clinical levels for enhanced assessment of NT-pro-BNP (CLEAN).. J Motor Learn Dev.

[7] Vaes B, Delgado V, Bax J (2010). Diagnostic accuracy of plasma NT-pro-BNP levels for excluding cardiac abnormalities in the very elderly.. BMC Geriatrics.

[8] Fonarow GC, Peacock WF, Phillips CO, Givertz MM, Lopatin M (2007). ADHERE scientific advisory committee and investigators. Admission B-type natriuretic peptide levels and in-hospital mortality in acute decompensated heart failure.. J Am Coll Cardiol.

[9] Palazzuoli A, Gallotta M, Quatrini I, Nuti R (2010). Natriuretic peptides (BNP and NT-proBNP): measurement and relevance in heart failure.. Vasc Health Risk Manag.

[10] Raizada A, Bhandari S, Khan MA (2007). Brain type natriuretic peptide (BNP) – a marker of new millennium in diagnosis of congestive heart failure.. Indian J Clin Biochem.

[11] Latini R, Masson S, Anand I (2004). The comparative prognostic value of plasma neurohormones at baseline in patients with heart failure enrolled in Val-HeFT.. Eur Heart J.

[12] Masson S, Latini R, Arnand IS, Phil D, Barlera S, Angelici L (2008). Prognostic value of changes in N-terminal proBNP in Val-HeFT.. J Am Coll Cardiol.

[13] Hartmann F, Packer M, Coats A (2004). Prognostic impact of plasma N-terminal pro-brain natriuretic peptide in severe chronic congestive heart failure: a substudy of the Carvedilol Prospective Randomized Cumulative Survival (COPERNICUS) trial.. Circulation.

[14] Castro LR, Alencar MC, Barbosa MM, Nunes Mdo C, Cardoso JR, Ribeiro AL (2011). NT-proBNP levels in patients with non-ST-segment elevation acute coronary syndrome.. Arq Bras Cardiol.

[15] Januzzi J, Camargo C, Anwaruddin S (2005). The N-terminal Pro-BNP Investigation of Dyspnea in the Emergency Department (PRIDE) Study.. Am J Cardiol.

[16] Booth J, Pinney J, Davenport A (2010). N-terminal pro-BNP – marker of cardiac dysfunction, fluid overload, or malnutrition in hemodialysis patients?. Clin J Am Soc Nephrol.

[17] Baig JA, Alam JM, Ansari MA (2010). Evaluation of NT pro BNP of diagnostic significance in patients with chronic kidney diseases.. Pak J Biochem Mol Biol.

[18] David S, Kumpers P, Seidler V (2008). Diagnostic value of N-terminal pro-B-type natriuretic peptide (NT-pro-BNP) for left ventricular dysfunction in patients with chronic kidney disease stage 5 on haemodialysis.. Nephrol Dial Transplant.

[19] Van der Horst ICC, de Boer RA, Hillege HL (2010). Neurohormonal profile of patients with heart failure and diabetes.. Netherlands Heart J.

[20] Andreas M, Zeisler H, Handisurya A (2011). N-terminal-pro-brain natriuretic peptide is decreased in insulin dependent gestational diabetes mellitus: a prospective cohort trial.. Cardiovasc Diabetol.

[21] Wong F (2009). Cirrhotic cardiomyopathy.. Hepatol Int.

[22] Henriksen JH, Gotze JP, Fuglsang S (2003). Increased circulating probrain natriuretic peptide (proBNP) and brain natriuretic peptide (BNP) in patients with cirrhosis: relation to cardiovascular dysfunction and severity of disease.. Gut.

[23] Kneiseler G, Herzer K, Marggraf G (2010). Die Interaktion zwischen Leber und Herz.. Z Herz-, Thorax- Gefäßchirurgie.

[24] Gallinat A, Neumann T, Kaiser G (2010). Brain natriuretic peptide (BNP): an early marker of cardiac dysfunction after liver transplantation?. Liver Outcomes, Supplement to Transplantation.

[25] Schreiber D, Nix DA, Brown DFM Natriuretic peptides in congestive heart failure.. http://emedicine.medscape.com/article/761722-overview.

